# Death from Chloroform

**Published:** 1865-01

**Authors:** 


Death from Chloroform.—An inquest has been held at the Bath United States Hospital on the body of John Downing, aged fifteen. Mr. R. T. Gore, surgeon of the hospital, said that the boy was admitted with a crippled leg, which no treatment could benefit. Amputation was resolved on, and on examination of the patient, made especially with reference to the heart's action, there appeared nothing to forbid the use of chloroform. The operation was successfully performed, but signs of failing circulation appeared, and death ensued in about ten minutes. Verdict: Died from the administration of chloroform through misadventure.—*Lancet*, 1864.

